# Origin of phase relative stability and phase transformation in an *S*-ibu­pro­fen–nicotinamide cocrystal

**DOI:** 10.1107/S2053229625008952

**Published:** 2025-10-21

**Authors:** Mathieu Guerain, Hubert Chevreau, Erik Elkaim, Yannick Guinet, Laurent Paccou, Florence Danède, Alain Hedoux, Frederic Affouard

**Affiliations:** a Université Lille, CNRS, INRA, ENSCL, UMR 8207 – UMET – Unité Matériaux et Transformations, F-59650 Villeneuve d’Ascq, France; b Soleil Synchrotron, L’Orme des Merisiers, Saint-Aubin, BP 48, 91192 Gif-sur-Yvette, France; Oak Ridge National Laboratory, USA

**Keywords:** crystal engineering, crystal structure, cocrystal, ibu­pro­fen, nicotinamide, powder X-ray diffraction, PXRD, SOLEIL Synchrotron, DFT

## Abstract

The crystal structure of the metastable form of *S*-ibu­pro­fen–nicotinamide cocrystals, C_13_H_18_O_2_·C_6_H_6_N_2_O, was solved from powder X-ray diffraction. This form was obtained by melting a molar mixture of *S*-ibu­pro­fen and nicotinamide at 100 °C, and then cooling. The high-resolution powder X-ray diffraction pattern of this new phase was recorded at room tem­per­a­ture using synchrotron radiation at SOLEIL Synchrotron (France). A hypothetical structure was ob­tained from the Monte-Carlo simulated annealing method and confirmed by Rietveld refinement. The symmetry is monoclinic (space group *P*2_1_, No. 4) and the unit cell contains four mol­ecules, two of nicotinamide and two of *S*-ibu­pro­fen. Density functional theory (DFT) energy minimization simulation was performed in order to locate the H atoms. The determination of the crystallographic structure of this metastable form allowed an explanation of the main mechanisms at the origin of the relative stability of the two forms of the *S*-ibu­pro­fen–nicotinamide cocrystals. This also made it possible to explain the transition mechanism between the two forms with tem­per­a­ture.

## Abstract 2

The crystal structure of the metastable form of *S*-ibu­pro­fen–nicotinamide cocrystals was solved from powder X-ray diffraction data and explains the main mechanisms responsible for the relative stability of the two forms of the cocrystals. This also made it possible to explain the transition mechanism between the two forms with tem­per­a­ture.

## Introduction

In recent years, the design of functional pharmaceutical mol­ecular materials by the cocrystallization technique has attracted increasing inter­est (Friščić & Jones, 2010 ▸) when other classical approaches based, for example, on salt formation or metastable polymorphs are not possible. Designing cocrystals is mainly based on the concepts used in crystal engineering (Desiraju, 2010 ▸), *i.e.* on the formation of supra­molecular hy­dro­gen-bonded networks *via* various hy­dro­gen-bond synthons. The stability of the supra­molecular organization is therefore highly dependant on the large variety of hy­dro­gen-bond strengths. Pharmaceutical cocrystals generally consist of an active pharmaceutical ingredient (API) and a coformer present in the same crystal structure (Friščić & Jones, 2010 ▸; Vishweshwar *et al.*, 2006 ▸; Schultheiss & Newman, 2009 ▸; Brittain, 2013 ▸; Childs *et al.*, 2009 ▸), for example, paracetamol–piperazine (Oswald *et al.*, 2002 ▸), ibu­pro­fen–nicotinamide (Berry *et al.*, 2008 ▸), carbamazepine–saccharin (Fleischman *et al.*, 2003 ▸), *etc*. These multicom­ponent materials in the crystalline solid state have an obvious inter­est in terms of stability, but also in improving many physicochemical properties of an API, such as aqueous solubility, dissolution, hygroscopicity or bioavailability (Higashi *et al.*, 2017 ▸; Friščić & Jones, 2010 ▸). However, the discovery and preparation of new cocrystals remain empirical, and still based on trial and error (ter Horst *et al.*, 2009 ▸). Cocrystallization can be achieved by many different techniques (Vishweshwar *et al.*, 2006 ▸; Karimi-Jafari *et al.*, 2018 ▸), such as crystallization in solution, grinding, grinding assisted by a solvent, use of supercritical fluids, sono­crystallization, *etc*., which may lead to different crystalline polymorphs in an uncontrolled manner (Schultheiss & New­man, 2009 ▸; ter Horst *et al.*, 2009 ▸). It is worth noting that using various cocrystallization synthesis methods should pro­mote various hy­dro­gen-bond types, and then various polymorphic or pseudopolymorphic forms of cocrystals, characterized by different degrees of stability.

**Figure 1:**
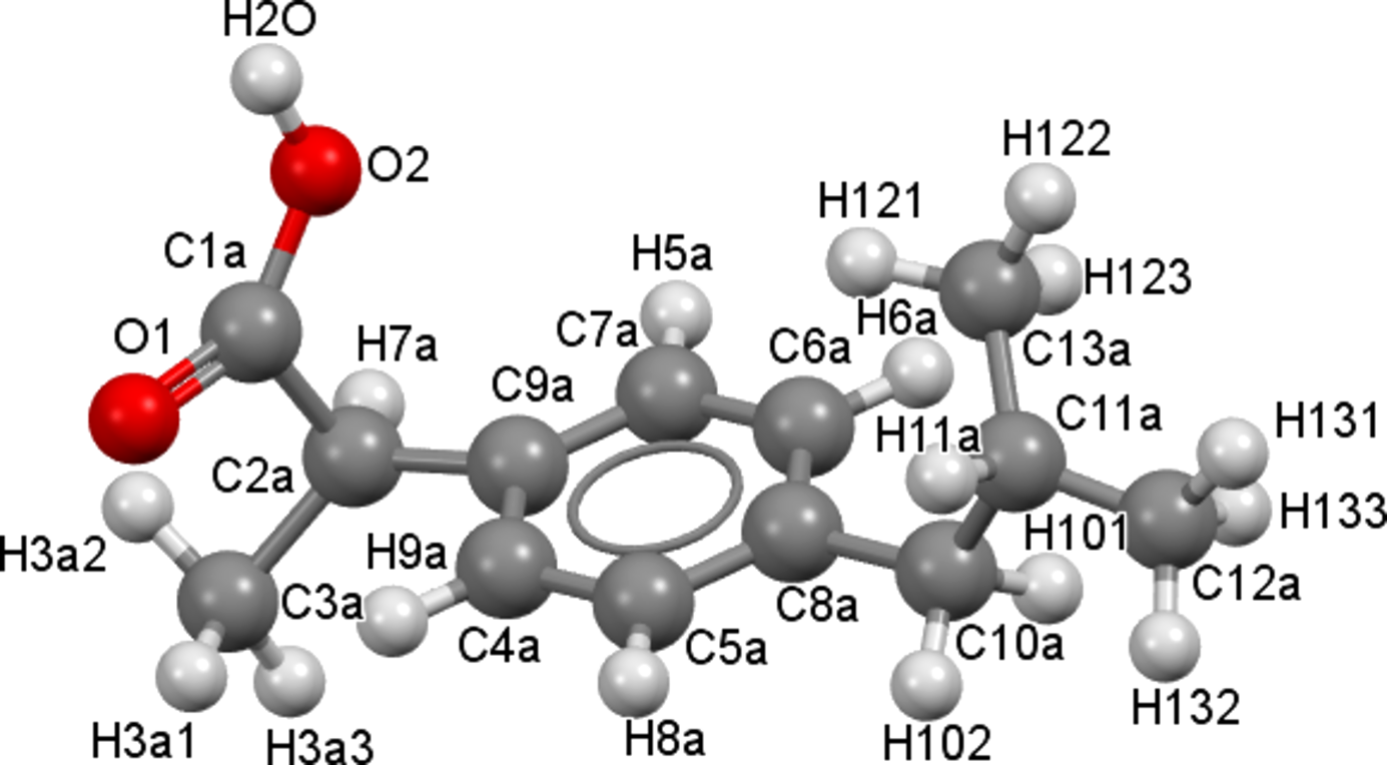
Representation of the ibu­pro­fen (IBP) mol­ecule. The atoms are labelled according to the CIF file for the present structure and Berry *et al.* (2008 ▸).

Ibuprofen (IBP, C_13_H_18_O_2_) is a well-known API used as a nonsteroidal anti-inflammatory drug (NSAID) to reduce fever and to treat pain or inflammation. It can be found in many formulations (powders, capsules, tablets, *etc*.) and is listed in the World Health Organization’s *Essential Drugs List* (WHO, 2007 ▸). Nevertheless, its low water solubility remains a challenge for oral use (Garzón & Martínez, 2004 ▸). Adding a second mol­ecule, and thus forming a cocrystal, to increase its solubility, is thus of inter­est. The crystallographic structure of *S*-ibu­pro­fen (*S*-IBP) is monoclinic (space group *P*2_1_) and its lattice parameters are *a* = 12.456 (3), *b* = 8.0362 (3), *c* = 13.533 (4) Å and β = 112.86 (3)° [Cambridge Structural Data­base (CSD; Groom *et al.*, 2016 ▸) refcode JEKNOC10 (Freer *et al.*, 1993 ▸)]. The melting point of *S*-ibu­pro­fen is 53 °C (Dwivedi *et al.*, 1992 ▸). The mol­ecule of *S*-ibu­pro­fen can be seen in Fig. 1 ▸.

Nicotinamide (N, C_6_H_6_N_2_O), one of the com­ponents of vitamin B, is currently used as a food additive. It is also one of the most popular coformers used for designing cocrystals, due to its high aqueous solubility and generally regarded as safe (GRAS) status.

**Figure 2:**
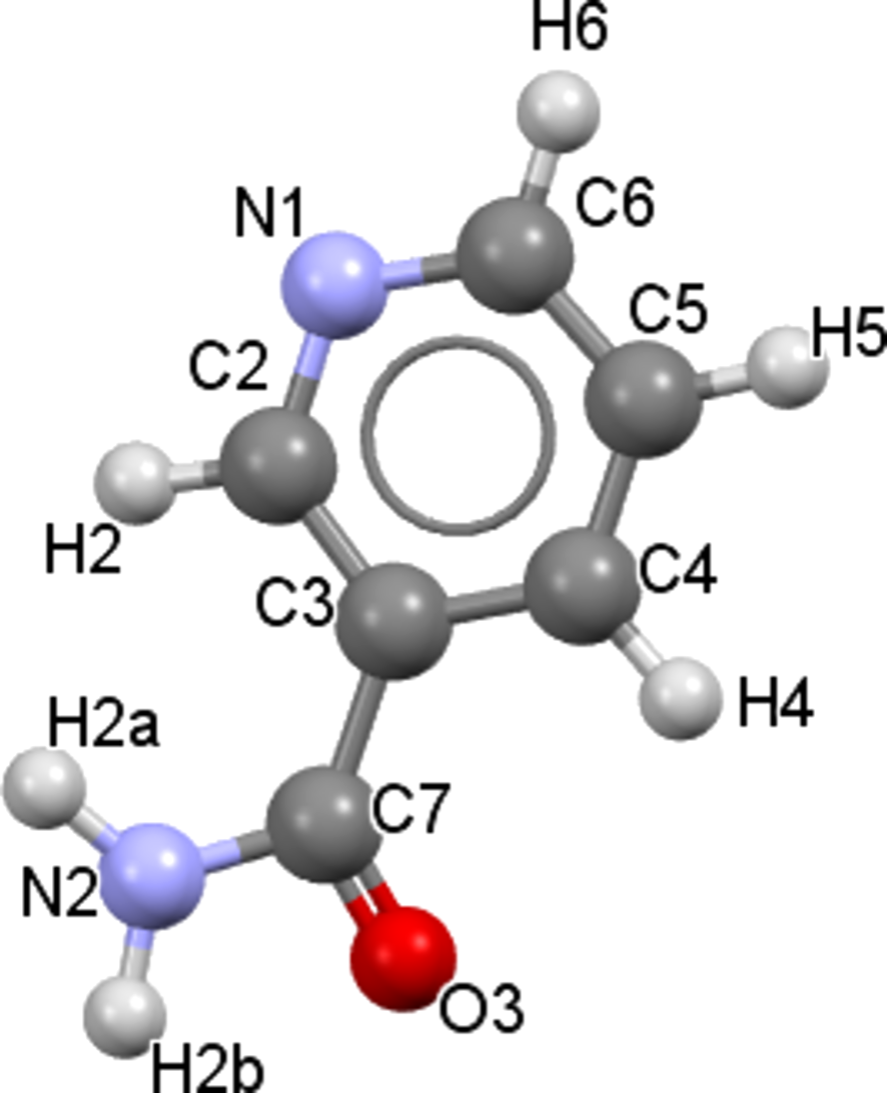
Representation of the nicotinamide (N) mol­ecule. The atoms are labelled according to the CIF file for the present structure and Berry *et al.* (2008 ▸).

Nicotinamide has a rich polymorphism with nine different polymorphs already known (Fellah *et al.*, 2021 ▸). The crystallographic structure of nicotinamide at atmospheric pressure and ambient tem­per­a­ture is monoclinic (space group *P*2_1_/*c*), and its lattice parameters are *a* = 3.975 (5), *b* = 15.632 (8), *c* = 9.422 (4) Å and β = 99.03 (7)° (NICOAM02 and NICOAM03; Miwa *et al.*, 1999 ▸). The mol­ecule of nicotinamide can be seen in Fig. 2 ▸.
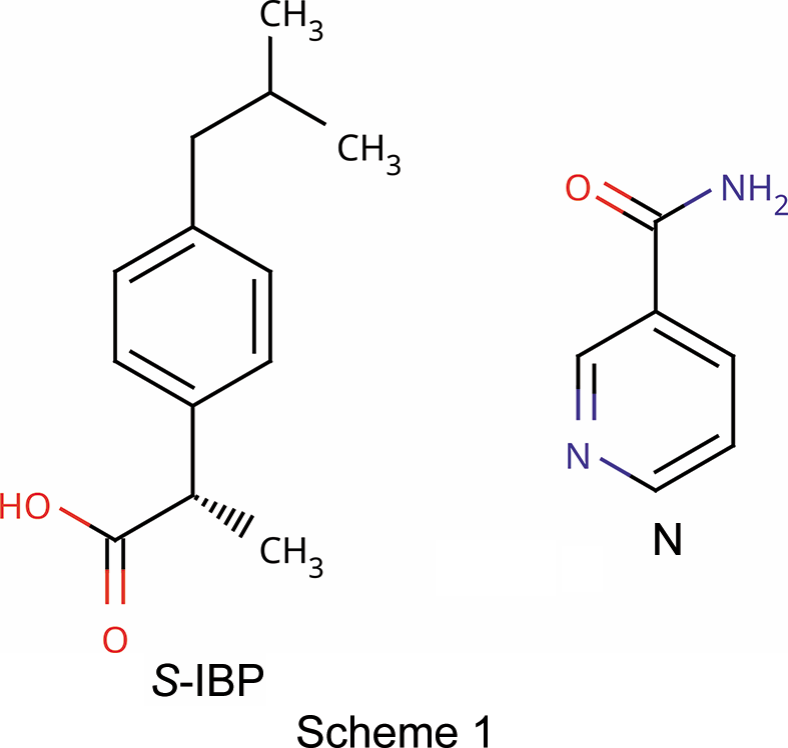


Cocrystals of *S*-ibu­pro­fen and nicotinamide (SOGLAC), and of *RS*-ibu­pro­fen and nicotinamide (SODDIZ) have been reported by Berry *et al.* (2008 ▸). The *RS*-ibu­pro­fen and nicotinamide cocrystal structure has also been confirmed by Alshahateet (2010 ▸). The synthesis of nicotinamide–ibu­pro­fen cocrystals provides the opportunity to combine the therapeutic effect of ibu­pro­fen with the high solubility of nicotinamide. The cocrystals of *S*-ibuprofen and nicotinamide obtained by Berry *et al.* (2008 ▸) crystallize in monoclinic sym­metry (space group *P*2_1_) and the lattice parameters are *a* = 5.4110 (6), *b* = 55.883 (6), *c* = 11.9006 (13) Å and β = 90.004 (2)°.

Recently, we reported a metastable form of this cocrystal, with a different crystallographic structure com­pared to that obtained by Berry *et al.* (2008 ▸), the metastable form being obtained from melting a mixture of *S*-IBP and N in equimolar proportions (Guerain, Guinet *et al.*, 2020 ▸). More specifically, cocrystals were synthesized using three different methods: (1) by milling, (2) by recrystallization from the melt and (3) by evaporation from a solution. Methods (1) and (3) lead to the already-known crystallographic structure of Berry *et al.* (2008 ▸), named form B. Method (2) leads to a new metastable crystallographic form of the cocrystal, named form A. After heating, form A is transformed into form B at 60 °C. Then, form B melts at 85 °C. The present article aims to resolve the structure of the new *S*-ibu­pro­fen–nicotinamide cocrystal (*S*-IBP:N, Scheme 1), obtained by melting the molar mixture, and to explain the mechanisms behind the polymorphic transition A→B with tem­per­a­ture and the relative stability between forms A and B. The structure was solved *ab initio* from powder X-ray diffraction using a direct-space approach (simulated an­neal­ing) and refined by the Rietveld method. The positions of the H atoms were estimated from density functional theory (DFT) energy minimization.

## Experimental

### Cocrystal synthesis

*S*-Ibuprofen (mol­ecular weight = 206.28 g mol^−1^) was pur­chased from Sigma–Aldrich (lot number BCBH0229V, purity 99%). Powder X-ray diffraction and Rietveld analysis have shown that the commercial material is in the monoclinic crystalline form (Freer *et al.*, 1993 ▸), as can be seen in Fig. S1 in the supporting information, and differential scanning calorimetry (DSC, Q1000, TA Instruments, 5 °C min^−1^) reveals the melting of the material at *T*_m_ = 53 °C.

Nicotinamide (mol­ecular weight = 122.12 g mol^−1^) was purchased from Sigma–Aldrich (lot number BCBV2931, purity >99.5%). Powder X-ray diffraction and Rietveld analysis have shown that the commercial material is in the most stable monoclinic crystalline *P*2_1_/*c* form (Miwa *et al.*, 1999 ▸), as can be seen in Fig. S1 in the supporting information, and differential scanning calorimetry (DSC, Q1000, TA Instruments, 5 °C min^−1^) reveals the melting of the material at *T*_m_ = 128 °C.

All the samples were analyzed without further purification.

The new cocrystal was obtained by the following methods.

An equimolar mixture (1:1), *i.e.* a mass of 61.89 mg of *S*-ibu­pro­fen and 36.63 mg of nicotinamide, was firstly melted at 100 °C, and secondly cooled:

– either to room tem­per­a­ture for isothermal crystallization

– or below the glass transition tem­per­a­ture, *T*_g_ = −20 °C (Guerain, Guinet *et al.*, 2020 ▸), for non-isothermal recrystallization upon heating from 25 °C (at a rate of 0.5 °C min^−1^).

Each time, the experiment was carried out both under a cover slip and in a capillary. The same results were obtained for both methods. In all cases, a new crystallographic form of *S*-IBP:N cocrystals was obtained (Guerain, Guinet *et al.*, 2020 ▸).

### Refinement

Crystal data, data collection and structure refinement details are summarized in Table 1 ▸. The powder X-ray dif­fraction patterns were measured at the Synchrotron SOLEIL, France, at the high-resolution powder diffraction beamline CRISTAL. The beamline is equipped with a 1D detector, ‘MYTHEN2 X’. The beamline was set up at an energy of 17 keV and then the energy was calibrated using NIST standard LaB6660a corresponding to a wavelength (λ) of 0.728302 Å. The cocrystal powder was enclosed in a glass capillary (diameter 0.5 mm) and mounted on the diffrac­tom­eter. The capillary was rotated during the experiments to improve the grain-sampling statistics. Data were collected at room temperature (∼20 °C) in the 1.2–40° 2θ range in 3 min.

The powder X-ray powder diffraction pattern of the new form of the cocrystal was com­pared to the most commonly used pattern databases for therapeutic materials. Coupling of the search/match functionalities of *Highscore* software with the CSD (Groom *et al.*, 2016 ▸), crystallographic open database (COD; Gražulis *et al.*, 2009 ▸) and the PDF-2 database of the Inter­national Center for Diffraction Data (ICDD) (Kabekkodu *et al.*, 2024 ▸) confirmed that this new form was not already known.

## Structure determination and Rietveld refinement

First, we com­pared the synchrotron pattern with the reference diffraction patterns obtained in the laboratory (Guerain, Guinet *et al.*, 2020 ▸). These observations showed that traces of pure nicotinamide remained in the sample analysed at the synchrotron. Indeed, as can be seen in Fig. S2 (see supporting information), the most intense peaks of pure nicotinamide can be observed on the cocrystal pattern between 6.8 and 6.9° and at 12.8°. Consequently, these peaks are not considered from the indexation stage.

For the indexation, the profiles of 20 reflections with a 2θ angle lower than 10° were refined individually with the programs *WinPlotr* (Roisnel & Rodríquez-Carvajal, 2001 ▸) and *DASH* (David *et al.*, 2006 ▸) in order to obtain their 2θ angular positions. The 2θ values of these reflections were com­puted in the programs *DICVOL* (Boultif & Louër, 2004 ▸), *TOPAS* (Markvardsen *et al.*, 2008 ▸; Coelho, 2000 ▸) and *McMaille* (Le Bail, 2004 ▸), and indexed. The best solution obtained with the three software programs has a monoclinic cell with the following parameters: *a* = 27.9997, *b* = 5.5302, *c* = 6.0231 Å, β = 93.1097° and *V* = 931.27 Å^3^. The calculated figures of merit are *M*_20_ = 26.7 and *F*_20_ = 92.6 (de Wolff, 1968 ▸; Smith & Snyder, 1979 ▸).

The experimental powder X-ray diffraction pattern was used from 1.2 to 24°. The model was refined using the Le Bail method (Le Bail *et al.*, 1988 ▸) within the program *DASH*. A pseudo-Voight function, a linear combination of a Lorentzian and a Gaussian of the same full width at half maximum (FWHM), was used to fit the Bragg peaks. This FWHM has a θ dependence according to Caglioti’s law (Caglioti *et al.*, 1958 ▸). The background was determined with a linear inter­polation between 50 points regularly distributed from 1.2 to 24°. The parameters were refined in the following order: the lattice parameters *a*, *b* and *c*, the zero-shift, the Caglioti profile parameters U, V and W, the mixing parameter η_0_ of the pseudo-Voight function and its 2θ dependence, and 50 points to define the background. Due to the high quality of the pattern, no parameters for the asymmetry of the Bragg peaks were used.

The *DASH* probabilistic approach to space-group determination, based on the systematic absences, was used and leads to the space group *P*2_1_ (No. 4) and a unit cell containing four mol­ecules. In order to obtain a starting structural model, the simulated annealing algorithm of the program *DASH* was used. The starting configuration of the *S*-IBP mol­ecule was borrowed from the monoclinic model (Freer *et al.*, 1993 ▸). The starting configuration of the N mol­ecule was borrowed from the monoclinic model (Miwa *et al.*, 1999 ▸). The mol­ecules were introduced randomly in the unit cell. The restraint options used for the calculations did not modify the bond lengths and bond angles. The translation and orientation parameters of the mol­ecule in the cell, as well as the torsion angles, were defined as variables in the calculation. From this structural model, rigid-body Rietveld refinement was performed using *DASH*. The refinement was performed in three steps: first, the global isotropic tem­per­a­ture factor, second, the translation and orientation parameters of the mol­ecule, and third, the torsion angles. The lattice parameters and the background parameters were set free.

Because H atoms are poorly located in powder X-ray powder diffraction data, such data were not used to independently refine the H-atom positions. They were optimized by density functional theory (DFT). The structural model was minimized using periodic density functional theory with fixed-cell dispersion-corrected density functional theory (DFT-D) (Giannozzi *et al.*, 2009 ▸, 2017 ▸). In this minimization, the positions of the heavy atoms were constrained, while the positions of the H atoms were let free. The Perdew–Burke–Ernzerhof (PBE) functional (Perdew *et al.*, 1996 ▸) was used with projector-augmented wave pseudopotentials and the Grimme D3 correction (Grimme *et al.*, 2010 ▸), as implemented in the pw.x executable of the *Quantum Espresso* program (Giannozzi *et al.*, 2009 ▸, 2017 ▸).

**Figure 3:**
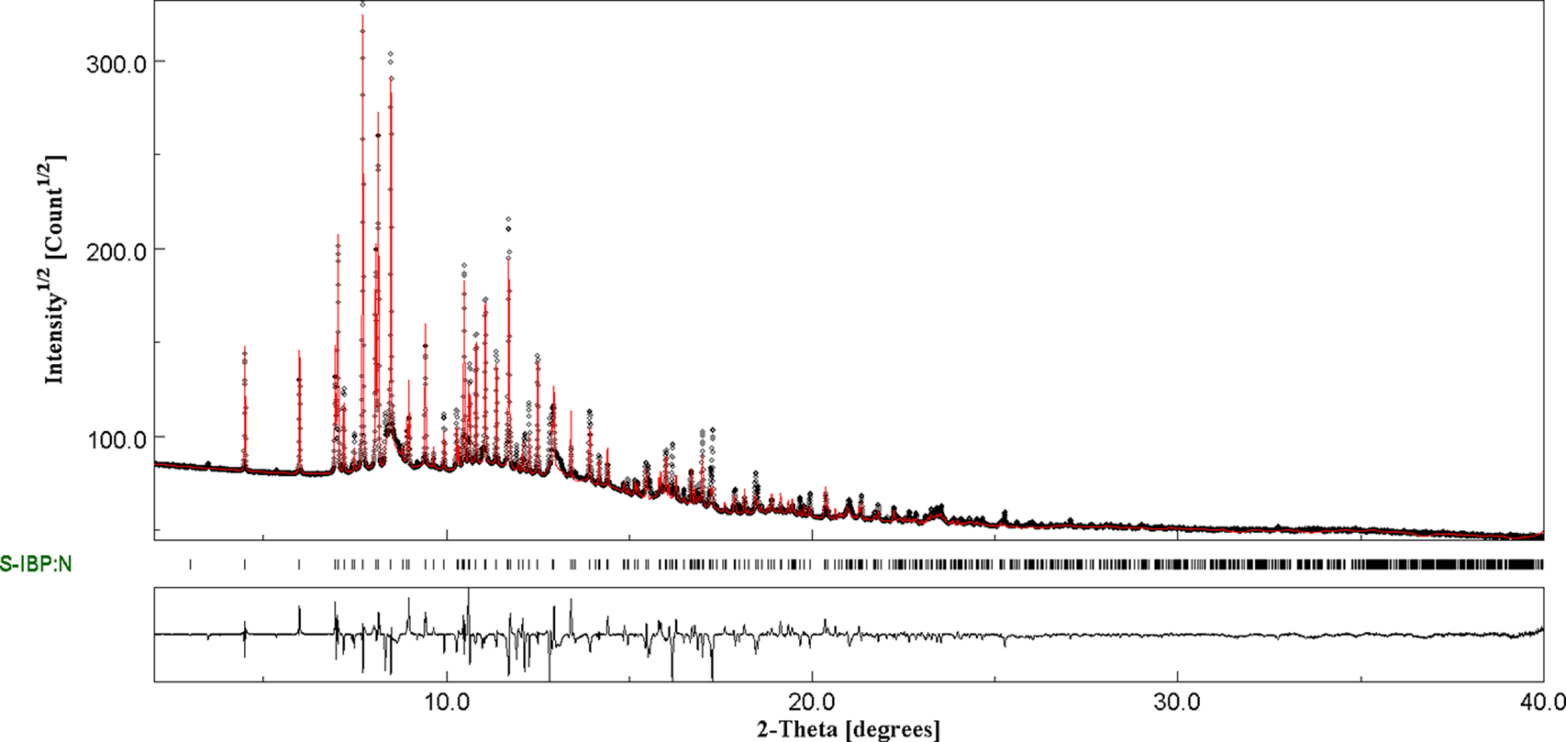
Final Rietveld plot of the *S*-ibu­pro­fen–nicotinamide cocrystal at room tem­per­a­ture. The observed intensities are indicated by dots and solid lines represent the best-fit profile (upper trace) and the difference pattern (lower trace). The vertical bars correspond to the positions of the Bragg peaks.

Then, from the structural model and the H-atom positions, atomic coordinates were introduced in the programs *MAUD* (Materials Analysis Using Diffraction) (Lutterotti, 2010 ▸) and *JANA2020* (Petrícek *et al.*, 2014 ▸), in order to graphically com­pare the calculated and experimental X-ray diffraction diagram (*MAUD*) and to generate the most accurate and com­plete CIF possible (*JANA2020*). The lattice parameters are *a* = 27.9925 (13), *b* = 5.5286 (2), *c* = 6.0213 (2) Å, β = 93.112 (2)° and *V* = 930.48 (7) Å^3^. The final conventional *R* factors are *R* = 0.0694, *R*_wp_(nb) = 0.0847, and *R*_exp _ = 0.007. The experimental and calculated diffraction patterns are shown in Fig. 3 ▸. Crystallographic data, profile and structural parameters are given in Table 1 ▸. The atomic positions from the final Rietveld refinement and DFT optimization can be seen in Table S1 in the supporting information. A com­parison between the refined and the DFT-optimized structure can also be seen in Fig. S3.

The *R* factors are com­parable to those obtained in the case of other cocrystals. In particular, similar factors are obtained with *R* = 0.1097 and *R*_wp_(nb) = 0.1547 for the *RS*-ibu­pro­fen–nicotinamide cocrystal, and *R* = 0.0582 and *R*_wp_(nb) = 0.138 for the *S*-ibu­pro­fen–nicotinamide form B cocrystal. It is also com­parable to the results obtained for the carbamazepine–tartaric acid cocrystal (Guerain, Derollez *et al.*, 2020 ▸) with the following *R* factors: *R* = 0.1207 and *R*_wp_(nb) = 0.1725. The correlation between the calculated and experimental X-ray diagram could be better in the absence of spurious peaks, probably due to the presence of nicotinamide impurities. However, calculations involving the stable form of nicotinamide have been performed but do not significantly improve this correlation. Unfortunately, due to the rich polymorphism of nicotinamide, it is not possible to know unambiguously the polymorphic form of nicotinamide present, and it is also possible that multiple polymorphs are present, which makes the calculation difficult.

## Discussion

**Figure 4:**
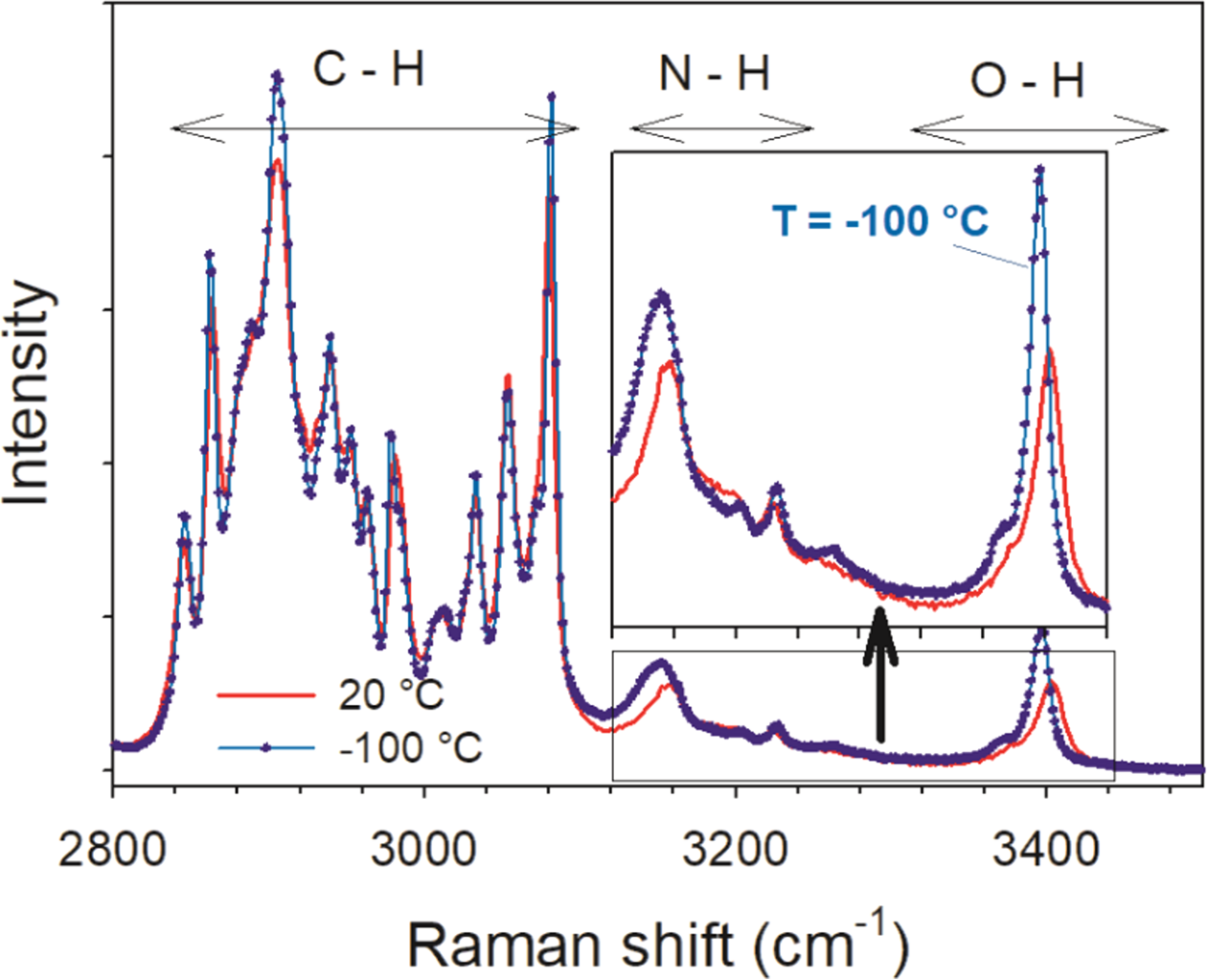
Intra­molecular C—H, N—H and O—H stretching region in form A at 20 and −100 °C (dashed line), and in Form B at 20 °C for *S*-IBP:N from Guerain, Guinet *et al.* (2020 ▸). The insert presents a zoom along the *y* axis of the N—H and O—H stretching region.

According to our previous experiments (Guerain, Guinet *et al.*, 2020 ▸), the Raman spectroscopy analysis performed on new *S*-IBP:N cocrystal form A clearly indicates both N—H⋯O mol­ecular associations and O—H⋯N associations. Fig. 4 ▸ shows the spectrum of *X*—H stretching vibrations (*X* = C, N and O) collected at 20 and −100 °C in a previous study (Guerain, Guinet *et al.*, 2020 ▸). It clearly shows three stretching vibrations of bonds involved in inter­molecular associations *via* hy­dro­gen bonding, easily recognized by the positive tem­per­a­ture dependence of the Raman bands. The N—H stretching band around 3150 cm^−1^ is distinctive of N—H⋯O mol­ecular associations, while the tem­per­a­ture dependences of O—H stretching bands located at 3390 and 3400 cm^−1^ reflect O—H⋯N associations.

**Figure 5:**
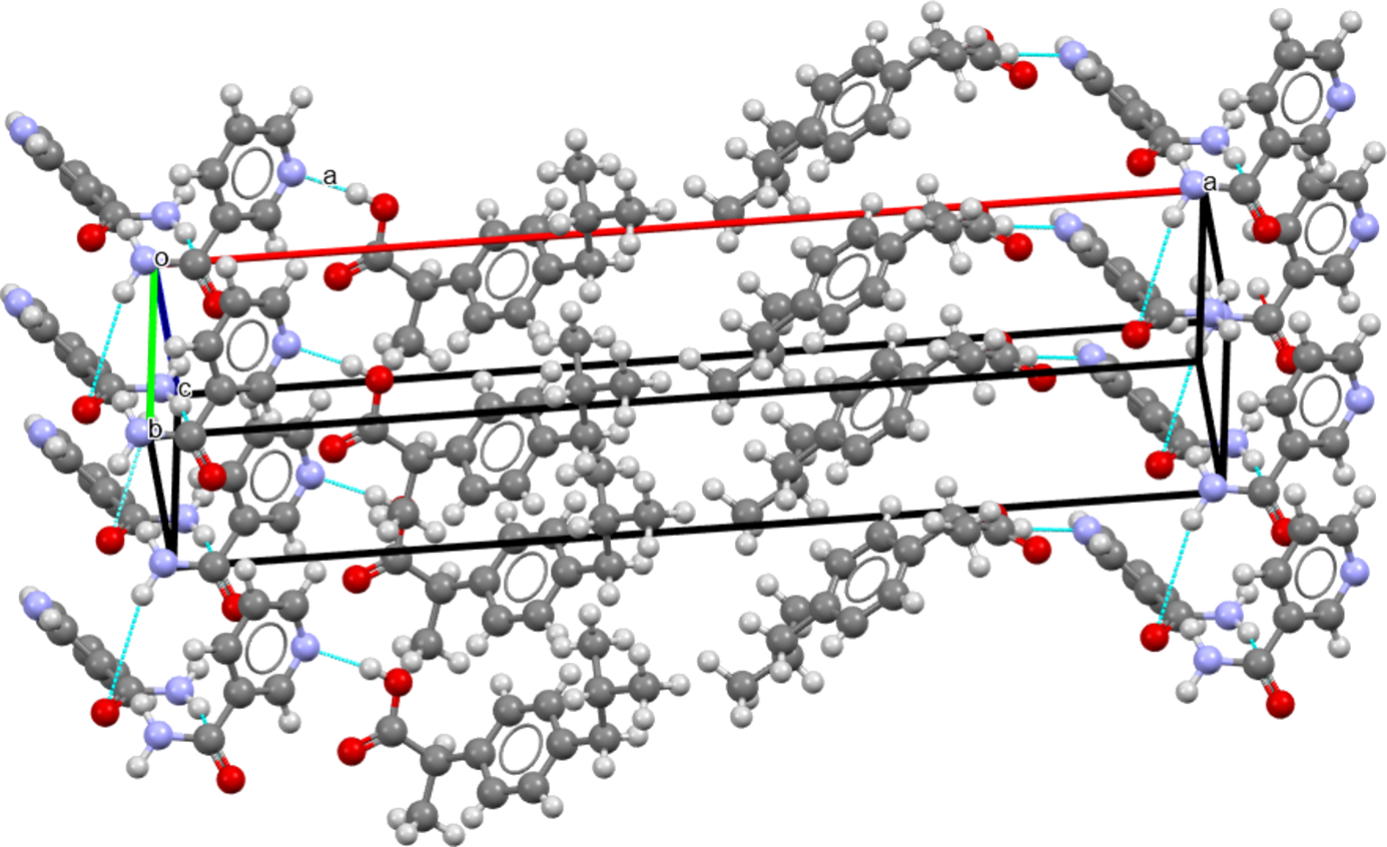
Projection of the unit cell along the [001] direction for *S*-ibu­pro­fen–nicotinamide cocrystal form A and visualization of the hy­dro­gen-bond network. Colour key: O atoms red, N atoms blue, C atoms black and H atoms white.

The crystallographic structure found here is consistent with these previous results, as can be seen in Fig. 5 ▸. Indeed, for the *S*-IBP:N cocrystal form A obtained here, N—H⋯O associations form between nicotinamide mol­ecules through their primary amide group. A nicotinamide mol­ecule is linked to two others by N—H⋯O hy­dro­gen bonds. The NH_2_ group of the mol­ecule is linked to the C=O groups of another nicotinamide mol­ecule and its own C=O group is linked to the NH_2_ group of a third nicotinamide mol­ecule. Moreover, O—H⋯N associations bind the ibu­pro­fen and nicotinamide mol­ecules through bonds between the N atoms of the nicotinamide pyridine ring and the H and O atoms of the ibu­pro­fen carboxyl group.

The structure of the new *S*-IBP:N cocrystal form A resolved in this work can also be com­pared to the already known (Berry *et al.*, 2008 ▸) cocrystal obtained by solvent evaporation or milling, namely form B (Guerain, Guinet *et al.*, 2020 ▸).

The lattice parameters of *S*-IBP:N cocrystal forms A and B are given in Table 2 ▸. Both structures exhibit a monoclinic symmetry and crystallize in the same space group *P*2_1_ (No. 4). Form B has a small lattice parameter (∼5 Å), an inter­mediate one (∼12 Å) and a larger one (∼56 Å), while form A has two small lattice parameter (∼5 and 6 Å) and a larger one (∼28 Å) which is half that of form B. This results in a division by four of the unit-cell volume for form B com­pared to form A reflecting the fact that the cell of form A contains two mol­ecules of ibu­pro­fen and two mol­ecules of nicotinamide, while form B contains eight mol­ecules of each. Moreover, there is a difference in the β angle of the two forms, the cocrystal obtained in this work having a β angle greater than that of form B, which is a little greater than 90°.

**Figure 6:**
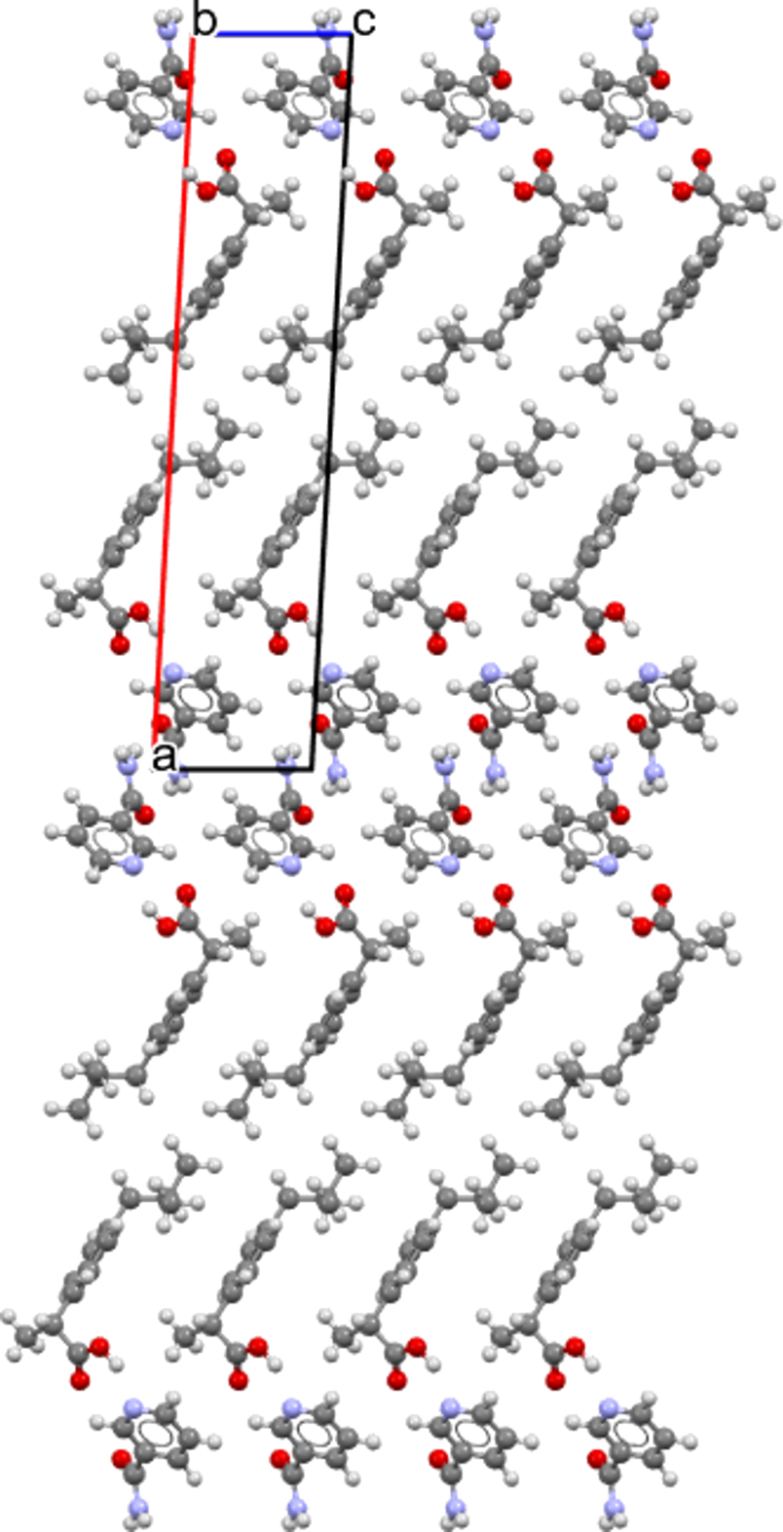
Projection of 4 × 2 cells of *S*-IBP:N cocrystal form A along the [010] direction.

These differences are due to a slight difference in the structural arrangement of the mol­ecules (see Figs. 6 ▸ and 7 ▸).

In both cocrystals, mol­ecules of ibu­pro­fen and mol­ecules of nicotinamide are stacked along the *c* axis without mirror projection or rotation from one mol­ecule to another. In cocrystal *S*-IBP:N form A, mol­ecules of ibu­pro­fen and mol­ecules of nicotinamide are also stacked along the *b* axis without mirror projection or rotation from one mol­ecule to another. Thus, in this cocrystal, lattice parameters *c* and *b* are similar in value.

In cocrystal *S*-IBP:N form B, mol­ecules of ibu­pro­fen and mol­ecules of nicotinamide are stacked along the *c* axis, as mentioned previously, and along the *a* axis without mirror projection or rotation from one mol­ecule to another.

The difference between the two cocrystals originates from the stacking of the mol­ecules along the larger axis (*a* for form A and *b* for form B). For cocrystal form A, along the *a* axis, there is an alternation of two mol­ecules of ibu­pro­fen, reversed with respect to each other, followed by two mol­ecules of nicotinamide, reversed with respect to each other, and so on. For cocrystal form B, there is also an alternation of two mol­ecules of ibu­pro­fen, reversed relative to each other, followed by two mol­ecules of nicotinamide, reversed relative to each other. However, the next two ibu­pro­fen mol­ecules are not in the same configuration as the two ibu­pro­fen mol­ecules preceding the nicotinamide mol­ecules. Indeed, there is a 180° rotation com­pared to the front one. The same is true for the nicotinamide mol­ecules, which are also rotated by 180° com­pared to the previous nicotinamide mol­ecules. Consequently, the *b* axis of *S*-IBP:N form B is twice that of the *a* axis of *S*-IBP:N form A. Thus, the structures of forms A and B are similar and differ mainly in the orientations of the benzene rings of ibu­pro­fen.

The small difference between the crystallographic lattice of form A and that of form B is in agreement with the observations made previously during differential scanning calorimetry (DSC) experiments (Guerain, Guinet *et al.*, 2020 ▸). The transformation A→B around 50 °C [revealed by Raman spectroscopy and X-ray diffraction in Guerain, Guinet *et al.* (2020 ▸)] was difficult to observe in DSC (see Fig. S4 in the supporting information). Thus, it had been hypothesized that this transformation during heating was subtle, with a low energy phase transition and fairly close crystallographic cell for forms A and B. In addition, such small differences between the crystal lattices of the two forms also lead to relatively similar diffraction patterns with a certain number of peaks of forms A and B located at the same positions. This point was also observed in our previous experiments (see Fig. S5 in the supporting information).

**Figure 8:**
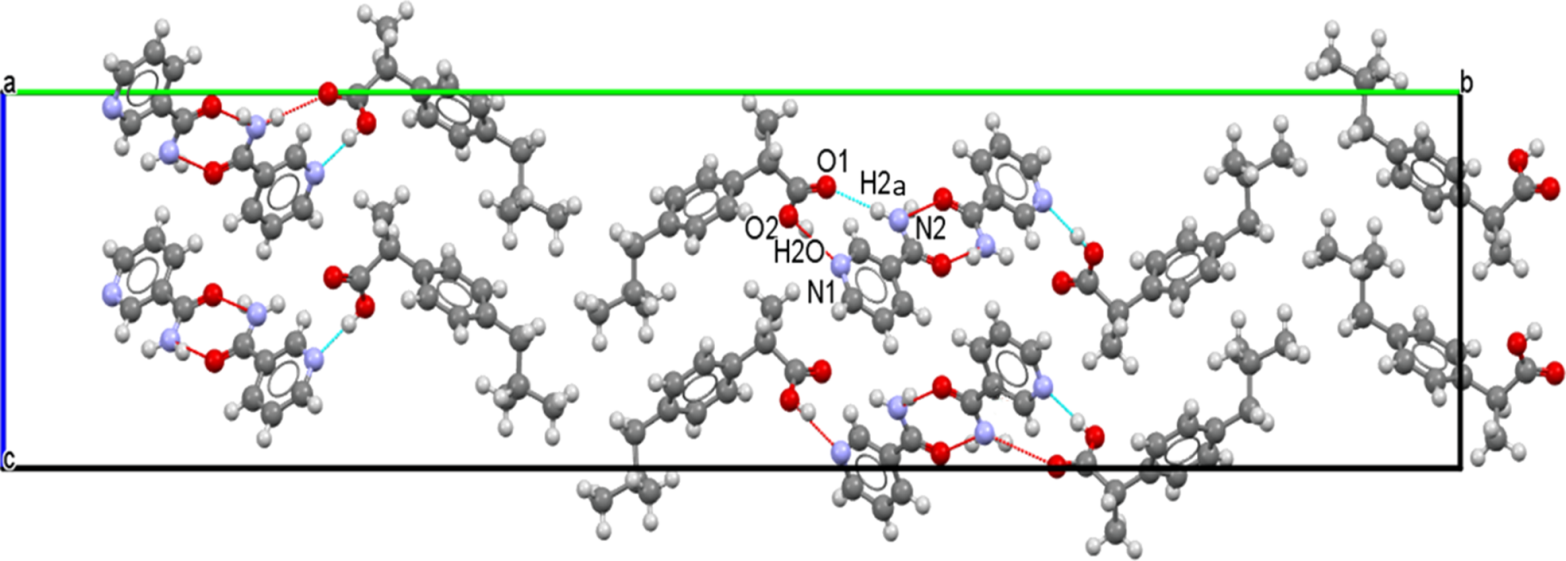
Projection of the unit cell along the [100] direction for *S*-ibu­pro­fen–nicotinamide cocrystal form B (Berry *et al.*, 2008 ▸) and visualization of the hy­dro­gen-bond network.

Regarding the cocrystal stability, we previously demonstrated that form A, which has been resolved here, is a metastable form, while form B is stable (Guerain, Guinet *et al.*, 2020 ▸). This relative stability and the transition from form A to form B can be explained by the com­parison between the structure obtained here and that obtained by Berry *et al.* (2008 ▸). For the structure of form A, as can be seen in Fig. 5 ▸, nicotinamide mol­ecules are linked together by N—H⋯O hy­dro­gen bonds. This is also true for form B, as can be seen in Fig. 8 ▸. However, for form A, one mol­ecule of nicotinamide is linked to two different nicotinamide mol­ecules by N—H⋯O hy­dro­gen bonds, whereas in the case of form B, two mol­ecules of nicotinamide are linked together by dimer associations. Moreover, the O2—H2O⋯N1 hy­dro­gen bond exists for both forms, but this bond has a shorter distance between O2 and N1 for form B than for form A (2.652 *versus* 2.689 Å). Therefore, this bond is more energetic and stable for form B. This result is in agreement with previously published Raman data (Guerain, Guinet *et al.*, 2020 ▸), where we showed that, when the tem­per­a­ture increases, the frequency of Raman bands O—H at 3375 cm^−1^ in form A is greater than in form B, reflecting weaker mol­ecular inter­actions between nicotinamide and ibu­pro­fen in form A than in form B. In this context, form A is less stable than form B, considering that hy­dro­gen bonds are responsible for the crystalline stability [the Raman spectra analysis performed and published in Guerain, Guinet *et al.* (2020 ▸) is given in Fig. S6 in the supporting information].

Finally, form A has a single bond between the mol­ecule of ibu­pro­fen and that of nicotinamide (Fig. 5 ▸), while form B has two (Fig. 8 ▸). Indeed, the O1⋯H2*A*—N2 hy­dro­gen bond does not exist in form A (see Figs. 5 ▸ and 8 ▸). Form B therefore has one more type of N—H⋯O hy­dro­gen bond than form A. In addition, the O—H⋯N hy­dro­gen bonds between the nicotinamide mol­ecules and the ibu­pro­fen mol­ecules are more energetic for form B than for form A. This justifies the different degrees of stability of the two forms observed previously (Guerain, Guinet *et al.*, 2020 ▸).

The polymorphic transition A→B could therefore be explained by the fact that in form A, under the effect of mol­ecular agitation, rotation of the –CONH_2_ group of nicotinamide would lead to the formation of a dimer on the one hand and to the appearance of a new N—H⋯O hy­dro­gen bond between the ibu­pro­fen mol­ecule and that of nicotinamide on the other hand. This new configuration associated with a shorter O—H⋯N hy­dro­gen bond would ensure a stronger inter­action between the nicotinamide and ibu­pro­fen mol­ecules, and would stabilize the structure into form B, the most stable form. However, van der Waals bonds also play an important role in the stability of cocrystals (Cruz-Cabeza *et al.*, 2024 ▸) and it would be inter­esting to carry out calculations involving them to confirm the stated hypothesis.

The energies of forms A and B as determined by the present study and in Berry *et al.* (2008 ▸) have been com­puted using periodic density functional theory employing the same methods as described in Section 3. As expected, metastable form A possesses a higher energy than stable form B. The energy difference is about 23.6 kcal mol^−1^, which is quite significant, but well in line with the trend reported in Nyman & Day (2015 ▸). It should be noted that this difference could be a little lower, since the structure of form A has been determined at *T* = 293 K, while form B was determined at *T* = 120 K.

## Supplementary Material

Crystal structure: contains datablock(s) global, I. DOI: 10.1107/S2053229625008952/vx3017sup1.cif

Structure factors: contains datablock(s) I. DOI: 10.1107/S2053229625008952/vx3017Isup2.hkl

PXRD patterns, DSC curves and comparison between refined Rietveld values and DFT-optimized coordinates. DOI: 10.1107/S2053229625008952/vx3017sup3.pdf

Supporting information file. DOI: 10.1107/S2053229625008952/vx3017Isup4.cml

CCDC reference: 2495679

## Figures and Tables

**Figure 7:**
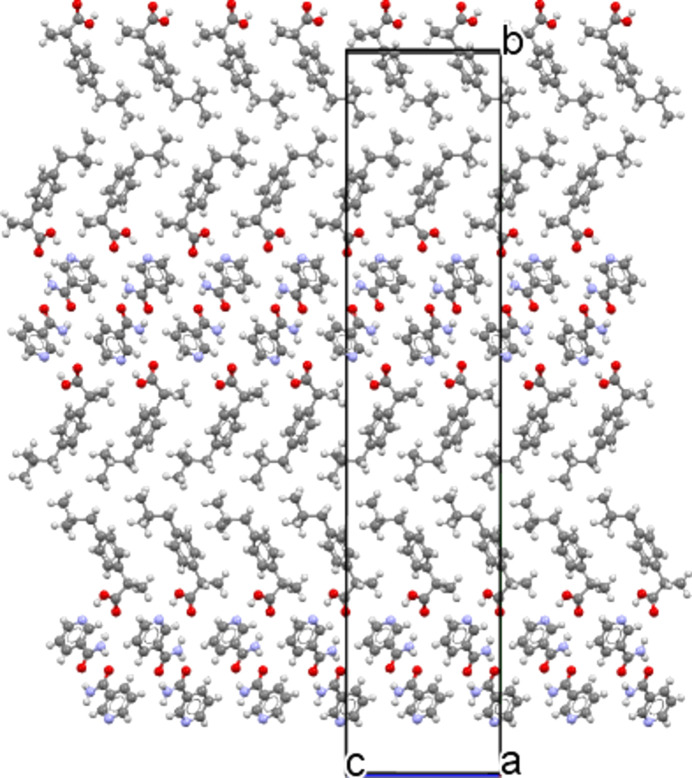
Projection of 4 × 1 cells of *S*-IBP:N cocrystal form B (Berry *et al.*, 2008 ▸) along the [100] direction.

**Table 1:** Crystallographic data, profile and structural parameters for *S*-ibu­pro­fen–nicotinamide cocrystal form A obtained after Rietveld refinement

Crystal data	
Chemical formula	C_19_H_24_N_2_O_3_
*M*_r_	328.4
Cell setting, space group	Monoclinic, *P*2_1_
Temperature (K)	293
*a*,*b*,*c* (Å)	27.9925 (13), 5.5286 (2), 6.0213 (2)
β (°)	93.112 (2
*V* (Å^3^)	930.48 (7)
*Z*	2
*F*(000)	352
μ (mm^−1^)	0.079
Specimen shape, size (mm)	Cylinder, 0.5
2θ range (°)	1.2–40.284°
	
Data collection	
Beamline	CRISTAL (SOLEIL)
Specimen mounting	0.5 mm diameter Lindemann capillary
Data collection mode	Transmission
Scan method	Continuous scan
Radiation type	Synchrotron 17 KeV, λ = 0.728302 Å
Binning size (°2θ)	0.004
	
Refinement	
*R* factors and goodness-of-fit	*R* = 0.0694, *R*_wp_(nb) = 0.0847, *R*_exp _ = 0.007

**Table 2:** Lattice unit-cell parameters com­parisons between *S*-ibu­pro­fen–nicotinamide cocrystal form A and *S*-ibu­pro­fen–nicotinamide cocrystal form B (Berry *et al.*, 2008 ▸)

Structure	*a* (Å)	*b* (Å)	*c* (Å)	β (°)	*V* (Å^3^)	Symmetry	Reference
*S*-IBP:N Form A	27.9925	5.5286	6.0213	93.112	930.48	*P*2_1_	This work
*S*-IBP:N Form B	5.4110	55.883	11.9006	90.004	3598.5	*P*2_1_	Berry *et al.* (2008 ▸)
