# Supplementary material for: Origin of phase relative stability and phase transformation in an S-ibu­pro­fen–nicotinamide cocrystal
Source: Acta Crystallogr C Struct Chem. 2025 Oct 21;81(Pt 11):658–65. doi: 10.1107/S2053229625008952 (PMC12587321; doi:10.1107/S2053229625008952)
Supplement: Supplementary file 3 [file c-81-00658-sup3.pdf]

Acta Cryst C

STRUCTURAL CHEMISTRY

Volume 81 (2025)

Supporting information for article:

Origin of phase relative stability and phase transformation in an *S*-ibuprofen–nicotinamide cocrystal

Mathieu Guerain, Hubert Chevreau, Erik Elkaim, Yannick Guinet, Laurent Paccou, Florence Danède, Alain Hedoux and Frederic Affouard

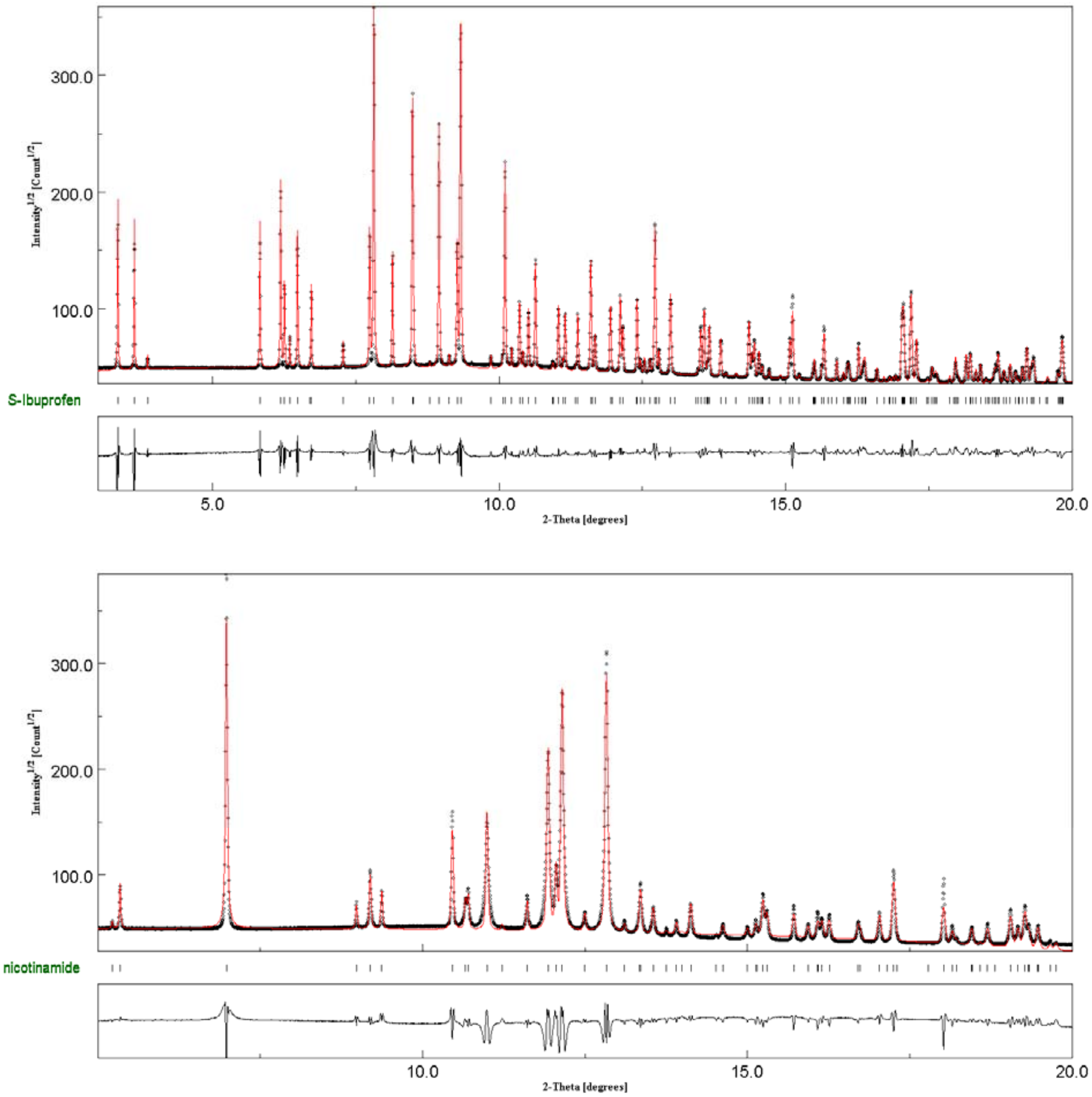

Figure S1 : Up: Rietveld plot of pure S-Ibuprofen at room temperature. Magnification between 5 and 20°. Down: Rietveld plot of pure Nicotinamide at room temperature. Magnification between 3 and 20°. Observed intensities are indicated by dots, and solid lines represent the best-fit profile (upper trace) and the difference pattern (lower trace). The vertical bars correspond to the positions of the Bragg peaks.

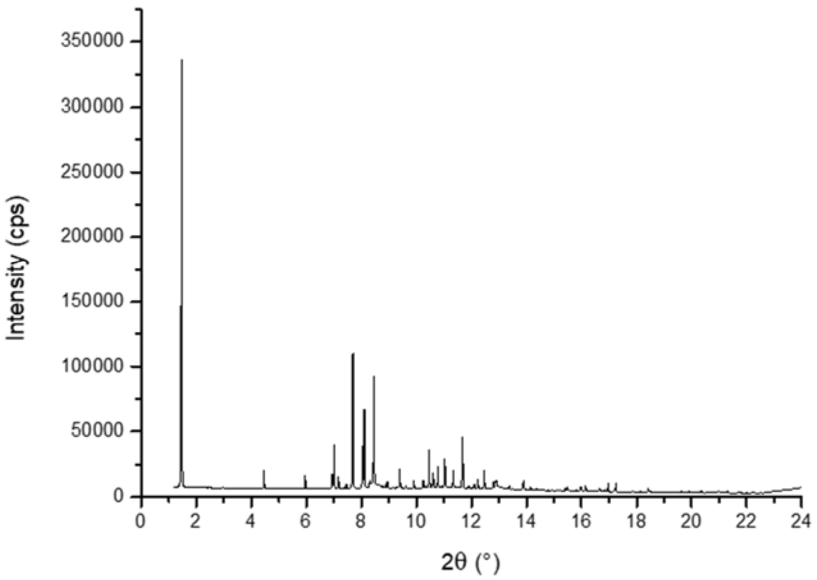
Intensity (cps)
2θ (°)
350000
300000
250000
200000
150000
100000
50000
0
0
2
4
6
8
10
12
14
16
18
20
22
24

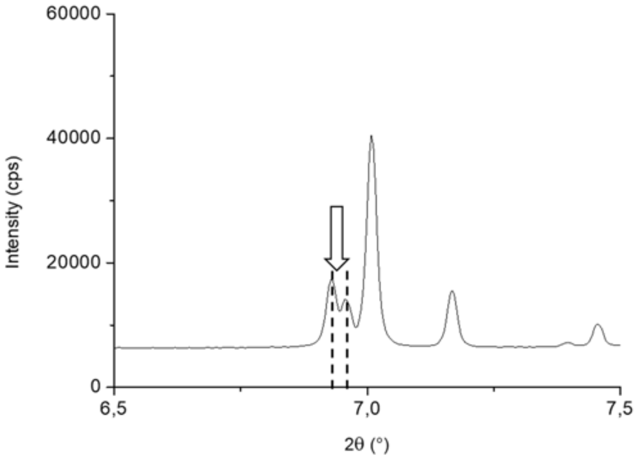
Intensity (cps)
2θ (°)
60000
40000
20000
0
6,5
7,0
7,5

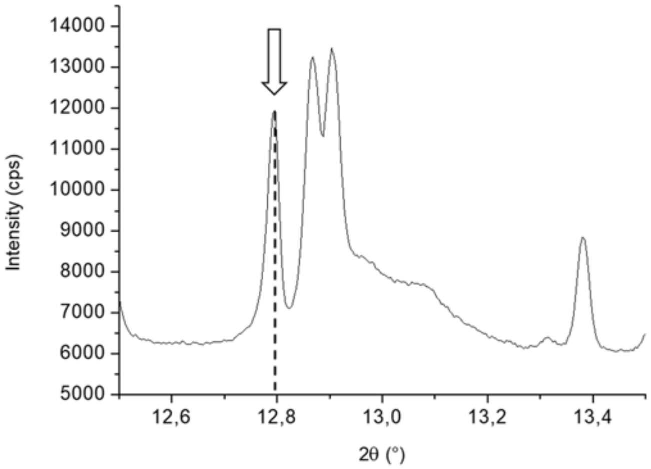

Figure S2: X-Ray Synchrotron diagram of S-IBP:N cocrystal (up), magnification between 6.5 and 7.5° (middle) and magnification between 12.5 and 13.5° (down). The arrow indicates where are the most intense peak of pure Nicotinamide. The dashed line indicates the peak which are not considered in the indexation.

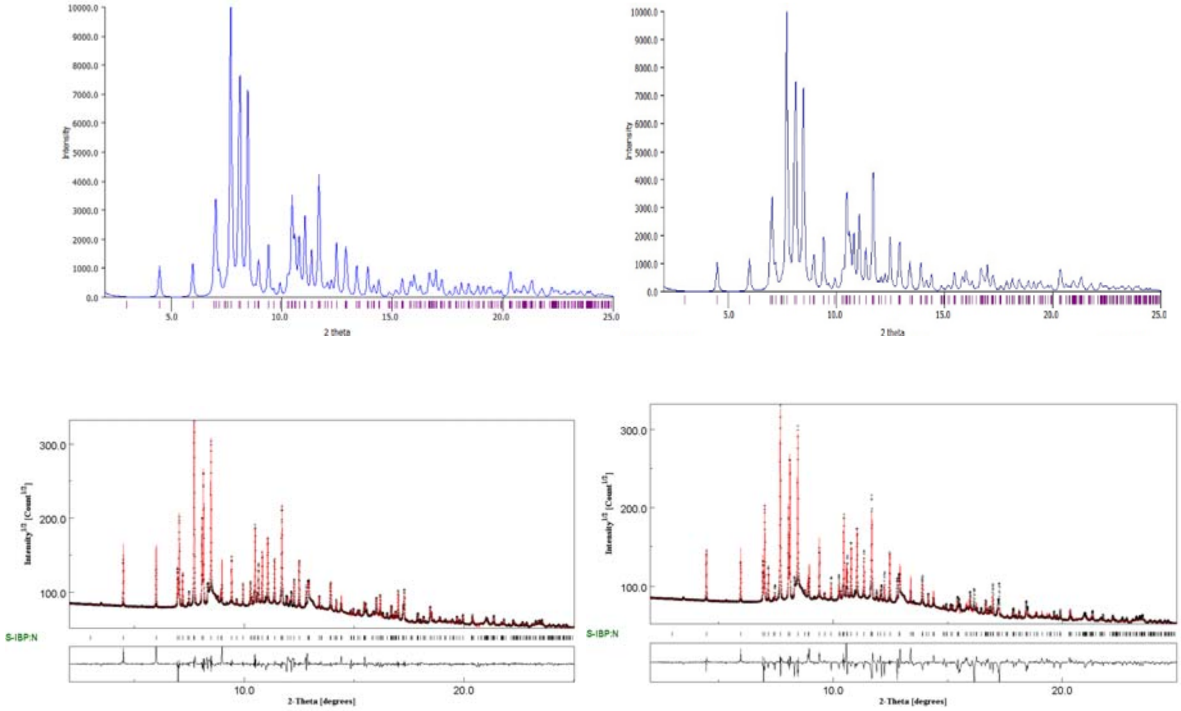

Figure S3: Calculation of the X-Ray diffractogram of S-IBP:N obtained with the refined structure (up left), Calculation of the X-Ray diffractogram of the S-IBP:N obtained with DFT optimization (up right), Rietveld plot of S-IBP:N obtained with refined structure (down left) and Rietveld plot of S-IBP:N obtained with DFT optimization (down right).

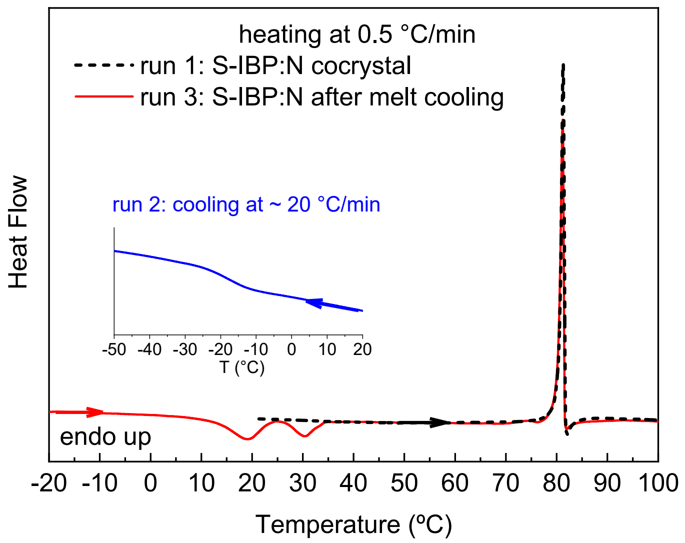

Figure S4 : DSC traces of S-IBP:N cocrystal after synthesis, melting and crystallisation from the melt. Run 1 and 3: heating at 0.5 °C /min.

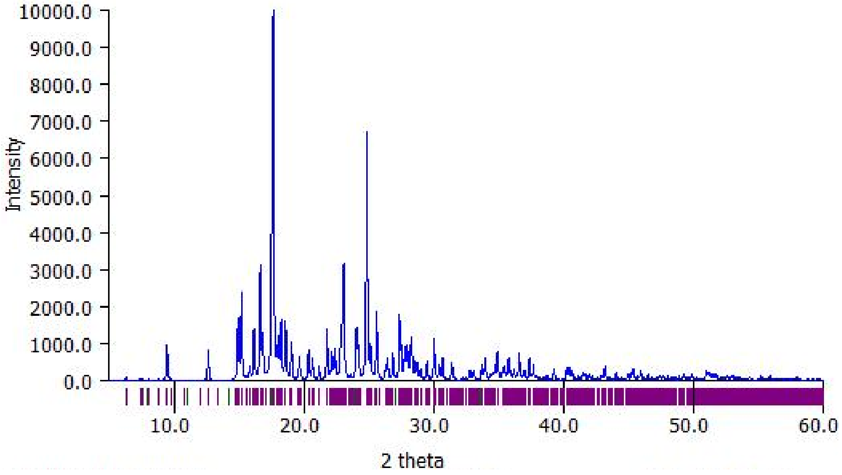

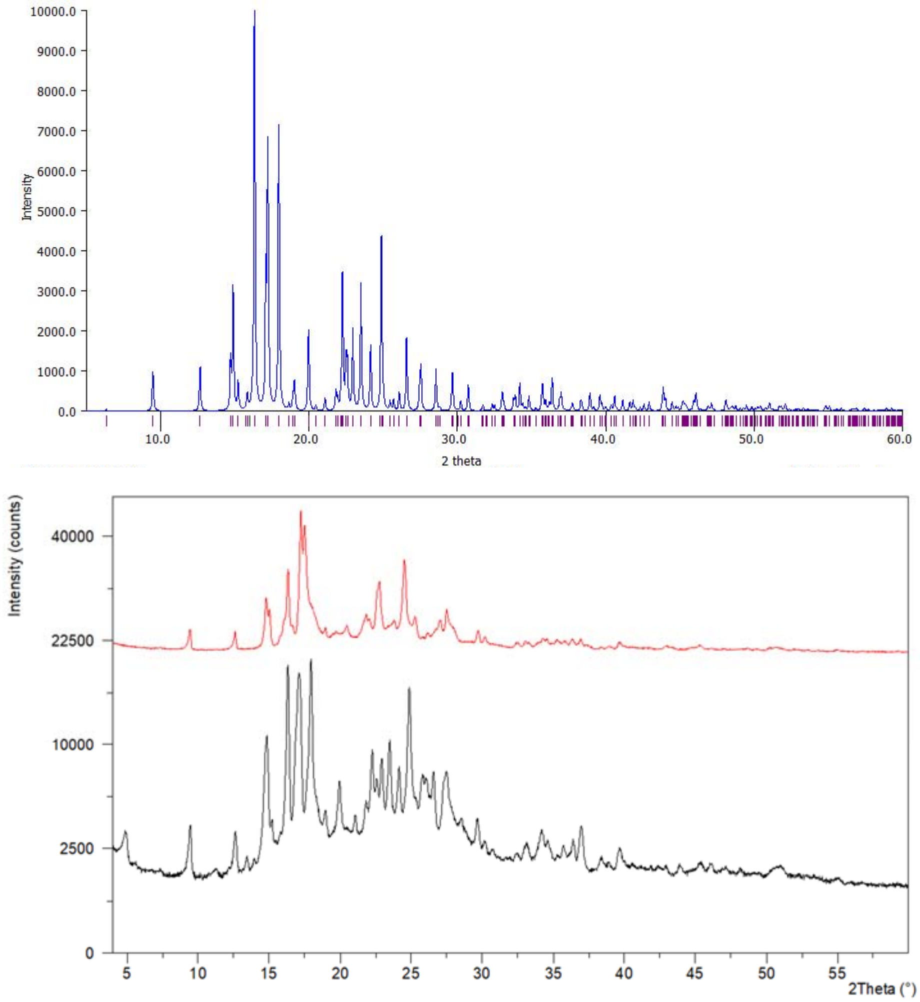

Figure S5 : up : Simulated X-Ray diagram of S-IBP:N Form B from (Berry *et al.*, 2008)*, middle : Simulated X-Ray diagram of S-IBP:N form A from this work, down : comparison of S-IBP-N Form B (red) and Form A (black) with laboratory setup from our previous work* (Guerain *et al.*, 2020)

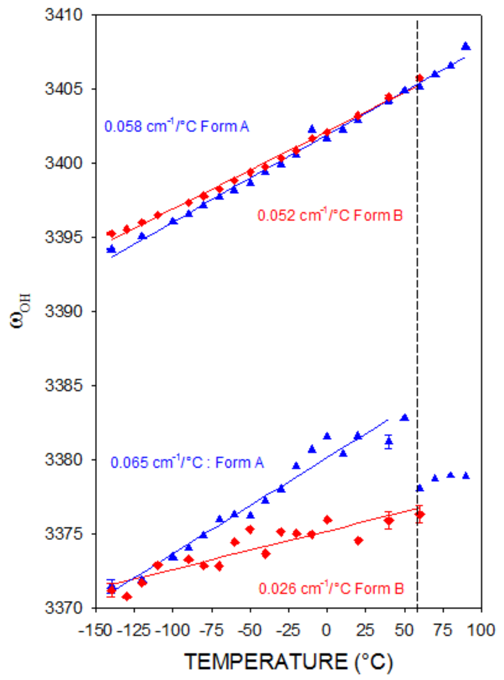

Figure S6 : Temperature dependence of O-H stretching band wavenumbers in Form A and B for S-IBP:N cocrystal. The lines correspond to the result of a least-square refinement procedure with a linear function of T. The values of slopes are reported in the graph. Triangles indicate Form A and diamonds indicates Form B.

Table S1 : Comparison between refined Rietveld values and DFT-optimized coordinates

Atom	refined values			DFT-optimized coordinates		
	X	Y	Z	X	Y	Z
C1	0.10542	0.78781	0.96105	0.10542	0.78781	0.96105
C2	0.0715	0.89643	0.81798	0.0715	0.89643	0.81798
C3	0.06348	0.79785	0.60733	0.06348	0.79785	0.60733
C4	0.09017	0.6005	0.5454	0.09017	0.6005	0.5454
C5	0.1233	0.50458	0.69961	0.1233	0.50458	0.69961
C6	0.04223	1.10996	0.87776	0.04223	1.10996	0.87776
N1	0.13085	0.59378	0.90493	0.13085	0.59378	0.90493
N2	-0.00513	1.09704	0.83783	-0.00513	1.09704	0.83783
O1	0.06198	1.28875	0.96245	0.06198	1.28875	0.96245
H1	0.11221	0.85118	1.12828	0.11236	0.86588	1.12274
H2	0.03549	0.87684	0.49651	0.04724	0.92338	0.50104
H3	0.08505	0.52005	0.38215	0.08937	0.52184	0.37897
H4	0.14473	0.35115	0.65726	0.14494	0.34914	0.65216
H5	-0.0212	0.94052	0.79667	-0.0165	0.95590	0.74238
H6	-0.02542	1.23961	0.88113	-0.01988	1.25882	0.79544
O2	0.16899	0.95336	0.25132	0.16899	0.95336	0.25132
O3	0.21438	0.65622	0.13975	0.21438	0.65622	0.13975
H7	0.46113	1.10245	-0.52283	0.46353	1.10804	-0.52395
H8	0.43613	0.73029	-0.15812	0.43445	0.72289	-0.14072
H9	0.46217	1.34307	-0.34301	0.46748	1.36087	-0.34559
H10	0.49269	1.08351	-0.24456	0.49336	1.08268	-0.26558

H11	0.25627	1.11609	0.72662	0.25759	1.12958	0.72312
H12	0.39447	1.18708	-0.32484	0.38667	1.17472	-0.32808
H13	0.44578	1.12008	0.07107	0.44887	1.10289	0.07969
H14	0.20411	1.03769	0.6767	0.19821	1.02048	0.70590
H15	0.41706	1.35289	-0.01217	0.42039	1.37417	0.00509
H16	0.37915	0.80001	-0.22382	0.37386	0.75769	-0.23209
H17	0.41041	0.7791	-0.42849	0.42052	0.75949	-0.42348
H18	0.36321	1.3927	0.20728	0.35758	1.45091	0.217857
H19	0.25054	0.75264	0.51399	0.25145	0.72202	0.533309
H20	0.38007	0.72943	0.07552	0.38339	0.72029	0.07826
H21	0.31025	0.63289	0.24243	0.30617	0.61096	0.23494
H22	0.28275	1.29856	0.36714	0.28130	1.34032	0.39502
H23	0.1904	0.65201	0.02652	0.18449	0.61626	0.04764
H24	0.21628	1.24069	0.53278	0.21710	1.24941	0.52134
C7	0.20574	0.83543	0.27205	0.20574	0.83543	0.27205
C8	0.24468	0.89242	0.44365	0.24468	0.89242	0.44365
C9	0.22863	1.08503	0.6075	0.22863	1.08503	0.6075
C10	0.28917	0.96305	0.3253	0.28917	0.96305	0.3253
C11	0.30315	1.20264	0.31605	0.30315	1.20264	0.31605
C12	0.34435	1.26828	0.21381	0.34435	1.26828	0.21381
C13	0.3731	1.09936	0.11913	0.3731	1.09936	0.11913
C14	0.35935	0.86131	0.13181	0.35935	0.86131	0.13181
C15	0.31843	0.79521	0.23264	0.31843	0.79521	0.23264
C16	C 0.41667	1.1768	-0.00151	C 0.41667	1.1768	-0.00151
C17	0.41689	1.08938	-0.24017	0.41689	1.08938	-0.24017
C18	0.41052	0.8192	-0.25505	0.41052	0.8192	-0.25505
C19	0.46232	1.16374	-0.34906	0.46232	1.16374	-0.34906
